# Trends in cervical cancer screening in Norway 2012–2017: a comparison study of non-immigrant and immigrant women

**DOI:** 10.1177/14034948231217636

**Published:** 2024-01-02

**Authors:** Marta Røttingen Enden, Kathy Møen, Jannicke Igland, Esperanza Diaz

**Affiliations:** Institute of Global Public Health and Primary Care, Faculty of Medicine, University of Bergen, Norway

**Keywords:** Cervical cancer, cancer screening, Norway, Scandinavia, emigrants and immigrants, population register, primary healthcare, uterine cervical neoplasms, health interventions

## Abstract

**Aims:**

Immigrant women in Norway have lower cervical cancer screening participation than non-immigrant women. Our aim in this study was to assess whether the observed increase in screening participation during 2012–2017 was different between Norwegian-born women and immigrant women.

**Methods:**

Data were collected from three national registries. The study included 1,409,561 women, categorized according to country of birth and immigrant background: (i) Norway, Norwegian parents; (ii) Norway, immigrant parent(s); (iii) Europe, excluding Norway; (iv) Africa; (v) Asia, including Turkey; and (vi) other countries. Trends and differences between groups were analyzed using Poisson regression analyses with adjustments for variables other studies have found to influence screening participation. Trends were assessed by including half-years as a continuous variable in the models and reported as prevalence ratios with 95% confidence intervals.

**Results:**

Screening participation increased in all groups, but was not statistically significant among women from Africa in the adjusted model. The highest increase was among Norwegian women, with a 2.2% increase per year. Interaction tests showed significantly smaller increases in screening among women born in Europe (*p* interaction < 0.0001), Africa (*p* interaction < 0.0001), Asia (*p* interaction < 0.0001), and countries in the “Other” category (*p* interaction = 0.004). There was also a smaller increase among Norwegian-born women with one or more immigrant parent(s), but this was not significant (*p* interaction = 0.178).

**Conclusions:**

**The gap in screening participation and the increasing differences in trends suggest that healthcare services do not reach all women in Norway to the same extent. One should attempt to improve this while working toward further increasing screening participation for all.**

## Introduction

Globally, cervical cancer is the fourth most common cancer among women, leading to more than 300,000 deaths annually [1]. Persistent infection with human papilloma virus (HPV) is the main risk factor for developing cervical cancer, and cancer development is highly preventable through vaccination and screening programs [2]. Cervical cancer screening including cervical cytology smears and HPV tests has been shown to reduce morbidity and mortality. The increase in detection of precancerous lesions and preinvasive cancer enables eradication before progressing to more aggressive cancer [3]. In Norway cervical cancer is the third most common cancer among women aged 25–49 years, and the twelfth most common cause of cancer mortality among women of all ages (3.6% of cancer mortality) [4].

Norway has universal healthcare coverage with funding predominantly from public sources. Patients have to pay a small out-of-pocket fee when they go to their general practitioners (GPs), including for screening [5]. Since the implementation of the national cervical cancer screening program in 1995, the incidence of and mortality from cervical cancer has declined in Norway [4]. From 2012 to 2017, the program sent invitation letters to all women between ages of 25 and 69 across Norway. These letters are a general invitation to get screened; the onus is on the women to make an appointment with their GP or another doctor. Women who have not been screened in the last 3 years receive a new letter, and an additional reminder is sent if no test is registered the following year [6].

Simultaneously with a positive trend in cervical cancer screening participation in Norway and other Scandinavian countries over the past years [7, 8], studies from several high-income countries have shown that immigrant women participate less in screening programs compared to non-immigrant women. These studies also highlighted the heterogeneity of immigrant women’s participation rates [9–12], and a recent systematic review and meta-analysis underlines this even further [13].

Immigrants and children born to one or two immigrant parent(s) comprised 13.1% of the Norwegian population in 2012 and 16.8% in 2017 [14]. Even though screening participation differs between non-immigrant and immigrant women across Scandinavia [11, 12, 15], no reports have looked into whether the positive trend in participation differs between groups.

A difference in trend could indicate that existing interventions are not equally effective in reaching all groups of women. If the positive trend is stronger among Norwegian-born women compared to women born outside of Norway, the group discrepancies could continue to increase. Such findings would imply that public health efforts related to increasing participation are not equitable. It is important to evaluate the effect of interventions to improve the screening participation among all women in Norway.

## Aims

The aim of this study was to assess whether the increase in cervical cancer screening participation differed between Norwegian-born women and various groups of immigrant women from 2012 to 2017.

## Methods

The study used data from three national registries: Statistics Norway, the Cancer Registry of Norway, and the Norwegian General Practitioners Register (GP database). To link the three registries, we used the unique personal identification number that all Norwegian citizens and legal immigrants residing in Norway for more than 6 months have. The study was approved by the Regional Committees for Medical and Health Research Ethics (2015/1156).

All women aged 25–69 years living in Norway at any point during 2012–2017 were eligible for inclusion in the study population (*N* = 1,684,981). Women in municipalities who had participated in an intervention study specifically targeting and encouraging immigrants to get screened for cervical cancer during the study period (*n* = 275,420) were excluded, as the aim of our study was to assess potential differences in trends in cervical screening participation if no specific interventions targeting migrants were conducted [16].

From Statistics Norway we acquired information on the women’s age, education level (low/no education or lower secondary school, middle/upper secondary school, high/university or college, and a category for those with missing data), household annual income (divided into terciles: low [less than NOK 430,000], middle [NOK 430,000–900,000], and high [more than NOK 900,000]), high- and medium/low level of urbanity for municipality of residence, and length of stay in Norway (less than 5 years, 5–10 years, 10–15 years, and more than 15 years). Level of urbanity was predefined into six categories by Statistics Norway with level 1 as the highest level of urbanity. The definition is based on a combination of population size and travel distances to workplaces and commercial services. In the current study we classified urbanity into two groups: level 1 and level 2–6. In order to have enough women in each category, we recategorized the original Statistics Norway’s classification groups into six mutually exclusive immigrant background categories based on their and their parents’ country of birth: (i) Norwegian-born women with two Norwegian parents, (ii) Norwegian-born women with one or two immigrant parent(s), (iii) women born in another country in Europe, (iv) women born in Africa, (v) women born in Asia, including Turkey, and (vi) women born in other countries (North, Central and South America, and Oceania).

Data from the Norwegian Cancer Registry provided information on women who had participated in the screening program and the date of their screening test(s). Each woman’s registered GP in 2017 was identified through the GP database and information on the GP’s gender and immigrant status (non-immigrant or immigrant) was obtained from Statistics Norway.

The age-standardized proportion of women participating in screening each half-year was calculated separately for each group according to migrant background, using direct standardization with the total population of women in Norway in 2012 in 5-year age groups as the standard population.

Differences in screening participation between groups were calculated using Poisson regression with Norwegian-born women with two Norwegian parents as the reference category. Before estimation of the Poisson models, data were aggregated into one observation per combination of 6-month period, screening status, 5-year age groups, and covariates with a count variable indicating the number of women within each aggregated observation. Each woman could only be represented once within each aggregated observation. The results were presented as prevalence ratios (PRs) with 95% confidence intervals. Different levels of adj-ustments were performed, adjusting for variables known to influence screening participation [15, 17]. Time in Norway was analyzed as a categorical variable because the association between time and log(rate) of participation was not linear. Model 1 adjusted for the women’s age and for the 6-month period in which the screening had taken place. Model 2 adjusted for the same characteristics as in Model 1 as well as level of education. Model 3a adjusted for the same characteristics as in Model 2 as well as level of urbanity for the municipality of residence and the characteristics of their GP (gender and immigration status). Model 3b adjusted for the same characteristics as Model 3a as well as for household occupational income. However, we did not have income data from 2017, thus this model only investigated 2012–2016. Most covariates were included as time-varying in the analyses, except information on GP characteristics, which was included as a fixed covariate with information from 2017.

Trends in cervical cancer screening participation were assessed with Poisson regression with calendar half-year as a continuous variable in the models (14 distinct periods of 6 months during 2012–2017). The PR for half-year was the measure for relative change in screening rates per half-year; for example a PR of 1.01 can be interpreted as 1% increase per half-year or 2% per year. The same levels of adjustments were carried out as described above (Models 1, 2, 3a, and 3b). We also created a Model 4 that excluded Norwegian women and adjusted for how long the immigrant women had lived in Norway. We tested for differences in screening participation trends between different groups based on country of birth by including an interaction term between continuous time (in half-years) and the categorical birth country variable with Norway as the reference. We used STATA version 16 for statistical analyses, and 5% was used as a significance level.

## Results

The study population included 1,193,461 women in the first half of 2012. In total, 1,409,561 distinct women were included in the study population for at least one half-year during 2012–2017. Table I presents the characteristics of the study population by background category in the first half of 2012. The mean age of the study population was 46.4 years (standard deviation (SD) 12.6) and was lower among immigrants compared to non-immigrants. Immigrants comprised 15.3% of the study population, with the largest group originating from Europe (7.5%). The mean length of stay in Norway was 15.1 (SD 14.2) years for women from Europe, 12.3 (SD 10.1) years for women from Africa, 15.1 (SD 10.9) years for women from Asia and Turkey, and 21.7 (SD 15.6) years for women from other countries.

**Table I.** Characteristics of the study population in the first half of 2012 (*N* = 1,293,461).

	Norway, Norwegian parents	Norway, immigrant parent(s)	Europe	Africa	Asia incl. Turkey	Others
Number of women	972,320	39,149	89,161	17,313	61,248	14,270
Age: mean (SD)	47.6 (12.6)	41.7 (11.8)	41.7 (11.8)	38.2 (9.4)	40.2 (10.5)	42.7 (11.2)
Level of education (%)						
Low	19.9	18.0	13.3	38.1	32.5	17.2
Middle	41.5	34.0	26.5	19.5	20.4	25.3
High	38.2	47.3	46.9	18.7	30.0	46.7
Missing	0.3	0.7	13.3	23.7	17.2	10.9
Household income (%)						
Low (NOK 0–430,000)	34.3	31.6	35.3	65.3	45.1	32.5
Middle (NOK 430.000–900,000)	33.8	35.7	38.7	24.3	35.9	35.4
High (NOK 900,000)	31.5	32.1	23.1	8.4	16.5	29.0
Missing	0.4	0.6	2.8	2.0	2.5	3.1
Municipality of residence (%)						
High level of urbanity	14.9	32.6	27.7	46.6	42.4	31.9
Medium/low level of urbanity	85.1	67.4	72.3	53.3	57.6	68.1
General practitioner’s gender (%)						
Male	52.6	50.9	48.6	52.0	45.6	51.0
Female	44.4	46.2	39.3	39.6	46.7	39.0
Missing	3.0	2.8	12.0	8.4	7.7	10.0
General practitioner’s origin (%)						
Born in Norway	69.4	65.8	52.7	50.1	50.8	60.3
Born abroad	28.2	32.0	38.9	44.6	43.4	32.9
Missing	2.4	2.1	8.4	5.3	5.8	6.8
Length of stay (%)						
Less than 5 years	-	-	35.7	30.8	24.1	19.8
5–10 years	-	-	15.9	23.1	19.3	14.0
10–15 years	-	-	11.8	18.5	15.3	9.9
More than 15 years	-	-	36.6	27.6	41.4	56.3

-: not applicable.

**Figure 1.**
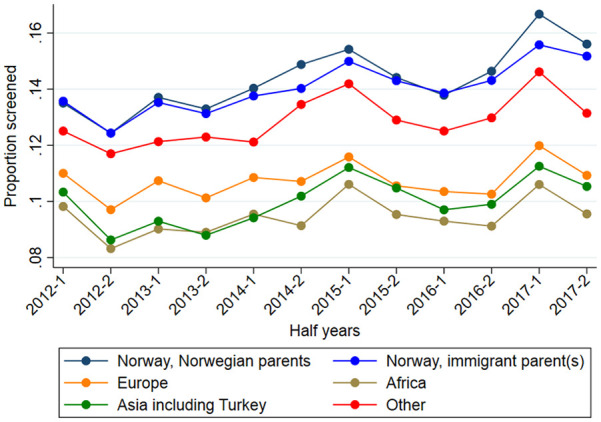
Age-standardized proportion of women getting screened in each half-year period, stratified by country of birth.

Regarding participation rates at the different time points, Figure 1 illustrates the age-standardized proportions of women getting screened in each half-year period, stratified by country of birth. Participation rates varied between the different groups, with higher participation among Norwegians with Norwegian parents and Norwegians with immigrant parent(s). When adjusting for age and half-year of calendar time in Poisson regression (Table II, Model 1) the participation was significantly lower in all groups compared to Norwegians with Norwegian parents. The largest difference was seen for women from Africa with 33% lower participation: PR (95% CI) = 0.67 (0.66–0.68). After adjustment for potential explanatory factors in Model 3a, the PR (95% CI) was reduced to 0.77 (0.76–9.78), still statistically significant.

**Table II.** Prevalence ratios (PRs) for screening participation for different immigrant groups compared to Norwegian women with Norwegian parents.

	Model^a^ PR (95% CI)	Model 2^b^ PR (95% CI)	Model 3a^c^ PR (95% CI)	Model 3b^d^ PR (95% CI)
Norway, Norwegian parents	1	1	1	1
Norway, immigrant parent(s)	0.963 (0.956–0.970)	0.962 (0.955–0.969)	0.952 (0.945–0.959)	0.965 (0.957–0.973)
Europe	0.723 (0.719–0.727)	0.767 (0.762–0.771)	0.786 (0.781–0.791)	0.806 (0.801–0.811)
Africa	0.672 (0.664–0.680)	0.774 (0.765–784)	0.769 (0.759–0.778)	0.818 (0.807–0.830)
Asia, including Turkey	0.711 (0.706–0.715)	0.779 (0.774–0.784)	0.785 (0.780–0.791)	0.810 (0.804–0.817)
Others	0.902 (0.891–0.913)	0.937 (0.926–0.949)	0.952 (0.940–0.964)	0.971 (0.957–0.985)

a Model 1: adjusted for women’s age and half-year of calendar time.

b Model 2: adjusted for women’s age, half-year of calendar time, and level of education.

c Model 3a: adjusted for variables in Model 2 plus additional adjustment for centrality, and the characteristics of their general practitioner (gender and background).

d Model 3b: adjusted for variables in Model 3 plus additional adjustment household occupational income group. 2017 is excluded from these analyses.

Regarding trends over time, as shown in Table III, the PR for time was >1 for each half-year in every category in all five models, indicating an increase in participation over time. However, the increase was not significant for women from Africa and Europe in Model 3b. The PR (95% CI) of 1.016 (1.015–1.016) in Model 3a indicated a 1.6% increase per half-year for Norwegians with Norwegian parents, or a 21.0% increase over 12 half-years if we assume a log-linear increase over time (e^ln(1.016)*12^ =1.210) (1.016^12=1.210). The trend in screening participation was similar for Norwegian women with immigrant parent(s), but the increase was smaller among women from other countries, particularly women from Africa.

**Table III.** Prevalence ratios per half-year increment in time for each immigrant group.

	Norwegians with Norwegian parents	Norwegians with immigrant parent(s)	Europe	Africa	Asia incl. Turkey	Other
	Prevalence ratio (95% confidence interval)					
Model 1^a^	1.017	1.016	*1.007*	1.006	1.013	1.012
	(1.017–1.018)	(1.014–1.018)	(1.006–1.009)	(1.003–1.010)	(1.011–1.015)	(1.009–1.016)
Model 2^b^	1.016	1.015	*1.012*	1.007	1.013	1.013
	(1.016–1.017)	(1.013–1.017)	(1.010–1.012)	(1.003–1.010)	(1.011–1.015)	(1.009–1.017)
Model 3a^c^	1.016	1.015	*1.008*	1.004	1.010	1.010
	(1.015–1.016)	(1.013–1.017)	(1.007–1.010)	(1.001–1.008)	(1.008–1.012)	(1.006–1.013)
Model 3b^d^	1.011	1.011	1.002	1.000	1.007	1.008
	(1.010–1.011)	(1.009–1.014)	(0.9996–1.004)	(0.996–1.005)	(1.005–1.010)	(1.003–1.013)
Model 4^e^			*1.005*	1.004	1.010	1.010
			(1.004–1.007)	(1.000–1.007)	(1.008–1.011)	(1.006–1.013)

a Model 1: adjusted for women’s age.

b Model 2: adjusted for women’s age and level of education.

c Model 3: adjusted for variables in Model 2 plus additional adjustment for centrality, and for the characteristics of their general practitioner (gender and background).

d Model 3b: adjusted for variables in Model 3 plus additional household occupational income group. 2017 is excluded from these analyses.

e Model 4: adjusted for variables in Model 3 plus the length of stay in Norway.

Interaction tests showed significantly smaller increases in cervical cancer screening participation among women born in Europe (*p* interaction < 0.0001), Africa (*p* interaction < 0.0001), Asia including Turkey (*p* interaction < 0.0001), and women born in countries belonging to the “Other” category (*p* interaction = 0.004) as compared to Norwegian women with two Norwegian-born parents (see Table III). Norwegian women with one or more foreign-born parent(s) also had a lower PR for the trend but the interaction term for interaction with time was not statistically significant (*p* interaction = 0.168).

## Discussion

There was an increase in cervical cancer screening participation from 2012 to 2017 among all women residing in Norway. However, the increase was significantly smaller among immigrant women compared to non-immigrant women, particularly am-ong those who originated from Europe, Africa, and Asia (including Turkey). For women born in Norway, the trend did not differ significantly be-tween women with two Norwegian-born parents and women with one or two immigrant parent(s). These findings were consistent when adjusting for factors that might impact screening participation, such as age, level of education, household income, urban/rural municipality of residence, and GP ch-aracteristics. In addition, we confirmed the previously described differences in participation in the cervical cancer screening among immigrants compared to Norwegian-born women at all time points throughout the study period.

There are various reasons for the gap in screening participation between immigrants and non-immigrants, and some of these can be alleviated [17, 18]. During the COVID-19 pandemic it became evident that national health information did not reach all groups residing in Norway to the same extent [19], and this may also be true for information on cancer screening. Since 2015, the Norwegian Cancer Society has held annual campaigns to increase overall cervical cancer screening participation. Although a general increase in participation could be a result of these campaigns, our data suggest that these campaigns did not influence immigrant women to the same extent as non-immigrant women, as the gaps between these groups seemed to remain or even broaden. The Norwegian Women’s Public Health Organization has held free “on the ground” campaigns targeting immigrant women. The first of these were organized in 2019, after our study period, and to the best of our knowledge there have been no systematic evaluations or reports from these efforts [20]. Future research should investigate how trends in cancer screening participation developed following these campaigns. Meanwhile, one could argue that the responsibility to provide equitable healthcare services should not only lie on the shoulders of non-governmental organizations, but also on the public healthcare system itself. Furthermore, local campaigns are costly, and catering to the heterogenicity of the immigrant population is challenging.

Previous studies have mainly investigated the differences in cervical cancer screening participation prevalence, and not differences in trends over time. These studies have identified substantial differences, also when adjusting for socioeconomic factors [10, 11, 21]. Some studies have attempted to go further into how these differences might be reduced, and intervention studies have been conducted both in Norway and other countries to increase screening participation [16, 22–24]. Some interventions have been culturally targeted to one or a few groups of immigrants[16, 24], while others have attempted to make health services more diversity-friendly [23]. A qualitative study from Oslo, the capital of Norway with a high percentage of immigrants, identified three communication strategies to increase participation among immigrants: 1) communication in-person at health centers, 2) communication through workshops and seminars to educate the women about the importance of screening, and 3) a better system of sending out reminders written in the native language of the women [18]. However, these strategies might be more challenging in the more remote areas or with women from countries with few immigrants. To increase participation among all groups, another strategy could be to educate healthcare professionals, increasing their cultural awareness and competence [23]. Both strategies, educating immigrant women in an appropriate manner and educating healthcare professionals, could be combined when attempting to improve cancer screening participation among immigrants [25].

Our data and analyses do not provide explanations for the differences in cancer screening participation among women of different geographic origins. Other studies have attempted to investigate this, and their results are likely to be applicable to our research setting. Potential explanations for differences in participation have been linked to cultural, economic, and structural barriers [17, 18]. Such barriers may also explain why trends in screening participation differ between women born in Norway and immigrant women. We did not find significant differences between women born in Norway, regardless their parents’ background, which differs from findings related to the use of oral contraception [26]. This discrepancy should be further studied.

Our study has several strengths and may be relevant for countries with similar population-based cervical cancer screening programs. The use of large national registry data sets allowed for various group comparisons. Furthermore, our study contributes to the gap in the existing literature as the trend, not just the prevalence, in cervical cancer screening participation was investigated and compared across different groups of women.

The results of our study may also have transferability to other cancer screening programs, such as colorectal cancer screening, which has been gradually implemented in Norway since May 2022 and targets people 55 years and older [27]. A qualitative study has already investigated factors that can be targeted to increase Polish immigrants’ access to the Norwegian colorectal cancer screening program. This study found that both cultural competence among healthcare providers and providing information in Polish could be effective measures when attempting to increase screening access [28]. Our study may also have transferability to the Norwegian mammography screening program which targets women between 50 and 69 years, as immigrant women have lower attendance rates in this program when adjusting for sociodemographic factors [29].

Our study has several limitations. Cancer screening guidelines recommend screening every 3 to 5 years, but our data spanned a period of 6 years and we therefore investigated trends over a limited period. Furthermore, some of the country-of-origin categories were heterogenous, and previous studies have found that non-adherence among immigrant women may vary substantially according to their country of origin [17]. However, dividing the categories into several subcategories to make them less heterogeneous would have led to small groups influencing the statistical power. The “Other” category, in particular, was perhaps too heterogenous to illustrate the actual cancer screening trends among women from North and South America and Oceania. The proximity to the women’s country of origin is of importance, as certain groups, such as women of European origin, may return to their birth country to get screened, particularly during their first years in Norway [17, 30]. This could imply an underestimated participation rate of these groups. However, length of stay in Norway did not seem to influence the trend in cancer screening participation (ref Model 4). Another limitation of our study was that we did not investigate to what extent women followed the recommended screening interval before or during the study period. We only investigated whether they had been screened during the half-year periods we created. Neither did we have data on how many times each woman had received invitation letters. It would have been interesting to sub-categorize the women into “screened within recommended time,” “ever screened,” and “never scr-eened” as previous screening participation often predicts future participation. Unfortunately, we did not have the data to do so.

Future research should analyze how the trends in cervical cancer screening participation have developed in the period after 2017 for the various groups. In addition, one should continue to investigate cancer screening interventions, both universal and those catering to specific groups, that can help narrow the gap between immigrant and non-immigrant women’s participation in cancer screening.

## Conclusion

Both the gap in cervical cancer screening participation prevalence and the differences in trends over time suggest that Norwegian healthcare services do not reach immigrant women to the same extent as Norwegian-born women.

## Supplemental Material

sj-docx-1-sjp-10.1177_14034948231217636 – Supplemental material for Trends in cervical cancer screening in Norway 2012–2017: a comparison study of non-immigrant and immigrant women Supplemental material, sj-docx-1-sjp-10.1177_14034948231217636 for Trends in cervical cancer screening in Norway 2012–2017: a comparison study of non-immigrant and immigrant women by MARTA RØTTINGEN ENDEN, KATHY MØEN, JANNICKE IGLAND and ESPERANZA DIAZ in Scandinavian Journal of Public Health
