# Supplementary material for: Trends in cervical cancer screening in Norway 2012–2017: a comparison study of non-immigrant and immigrant women
Source: Scand J Public Health. 2024 Jan 2;52(8):927–33. doi: 10.1177/14034948231217636 (PMC11626844; doi:10.1177/14034948231217636)
Supplement: sj-docx-1-sjp-10.1177_14034948231217636 – Supplemental material for Trends in cervical cancer screening in Norway 2012–2017: a comparison study of non-immigrant and immigrant women [file sj-docx-1-sjp-10.1177_14034948231217636.docx]

|  | Norway. Norwegian parents | Norway. Immigrant parent(s) | Europe | Africa | Asia incl. Turkey | Others |
| --- | --- | --- | --- | --- | --- | --- |
| Number of women | 962,504 | 48,931 | 119,265 | 25,503 | 79,882 | 17,115 |
| Age [mean (SD)] | 47.6 (12.8) | 41.4 (12.6) | 41.6 (11.4) | 39.3 (9.9) | 41.7 (10.7) | 43.9 (11.5) |
| Level of education (%) | | | | | | |
| Low | 17.7 | 17.0 | 11.2 | 37.6 | 29.1 | 16.2 |
| Middle | 38.0 | 30.0 | 21.5 | 17.9 | 19.4 | 23.2 |
| High | 44.0 | 52.1 | 43.3 | 18.1 | 30.7 | 46.4 |
| Missing | 0.3 | 0.9 | 24.0 | 26.5 | 20.9 | 14.3 |
| Household income (%) | | | | | | |
| Low (0-430,000NOK) | 0 | 0 | 0 | 0 | 0 | 0 |
| Middle (430,000-900,000NOK) | 0 | 0 | 0 | 0 | 0 | 0 |
| High (900.000-NOK) | 0 | 0 | 0 | 0 | 0 | 0 |
| Missing | 100 | 100 | 100 | 100 | 100 | 100 |
| Municipality of residence (%) | | | | | | |
| High level of urbanity | 15.1 | 35.0 | 27.0 | 40.0 | 38.2 | 32.1 |
| Medium/low level of urbanity | 84.9 | 65.0 | 74.1 | 60.0 | 61.9 | 67.9 |
| General practioner's gender (%) | | | | | | |
| Male | 52.4 | 50.8 | 55.2 | 55.1 | 48.5 | 55.6 |
| Female | 46.5 | 48.5 | 42.2 | 43.2 | 49.4 | 42.8 |
| Missing | 1.1 | 0.7 | 2.6 | 1.8 | 2.1 | 1.6 |
| General practioner’s origin (%) | | | | | | |
| Born in Norway | 70.0 | 66.0 | 55.1 | 51.8 | 52.5 | 62.9 |
| Born abroad | 28.5 | 33.0 | 42.0 | 46.1 | 45.2 | 35.3 |
| Missing | 1.4 | 1.0 | 2.9 | 2.1 | 2.4 | 1.8 |
| Length of stay (%) | | | | | | |
| Less than 5 years | - | - | 26.4 | 25.8 | 21.1 | 16.6 |
| 5-10 years | - | - | 27.5 | 25.8 | 19.4 | 15.4 |
| 10-15 years | - | - | 11.8 | 16.9 | 15.4 | 11.5 |
| More than 15 years | - | - | 34.4 | 31.5 | 44.0 | 56.5 |
| Missing |  |  | 0.0 | 0.0 | 0.0 | 0.0 |

***Table S1:* Characteristics of the study population in the second half of 2017 (n=1,253,200)**
